# A Versatile Bioreactor for Dynamic Suspension Cell Culture. Application to the Culture of Cancer Cell Spheroids

**DOI:** 10.1371/journal.pone.0154610

**Published:** 2016-05-04

**Authors:** Diana Massai, Giuseppe Isu, Denise Madeddu, Giulia Cerino, Angela Falco, Caterina Frati, Diego Gallo, Marco A. Deriu, Giuseppe Falvo D’Urso Labate, Federico Quaini, Alberto Audenino, Umberto Morbiducci

**Affiliations:** 1 Department of Mechanical and Aerospace Engineering, Politecnico di Torino, Turin, Italy; 2 Department of Clinical and Experimental Medicine, Università degli Studi di Parma, Parma, Italy; 3 Istituto Dalle Molle di studi sull'Intelligenza Artificiale, Scuola universitaria professionale della Svizzera italiana, Università della Svizzera italiana, Manno, Switzerland; 4 Bioexpansys Srl, Turin, Italy; Centro Cardiologico Monzino, ITALY

## Abstract

A versatile bioreactor suitable for dynamic suspension cell culture under tunable shear stress conditions has been developed and preliminarily tested culturing cancer cell spheroids. By adopting simple technological solutions and avoiding rotating components, the bioreactor exploits the laminar hydrodynamics establishing within the culture chamber enabling dynamic cell suspension in an environment favourable to mass transport, under a wide range of tunable shear stress conditions. The design phase of the device has been supported by multiphysics modelling and has provided a comprehensive analysis of the operating principles of the bioreactor. Moreover, an explanatory example is herein presented with multiphysics simulations used to set the proper bioreactor operating conditions for preliminary *in vitro* biological tests on a human lung carcinoma cell line. The biological results demonstrate that the ultralow shear dynamic suspension provided by the device is beneficial for culturing cancer cell spheroids. In comparison to the static suspension control, dynamic cell suspension preserves morphological features, promotes intercellular connection, increases spheroid size (2.4-fold increase) and number of cycling cells (1.58-fold increase), and reduces double strand DNA damage (1.5-fold reduction). It is envisioned that the versatility of this bioreactor could allow investigation and expansion of different cell types in the future.

## Introduction

The large scale production of cells is a mandatory step to set up economically viable in vitro experimental models for basic research, disease modelling and drug testing, and to definitely translate tissue engineering and regenerative medicine strategies to the clinical practice for therapeutic applications. However, scalability and standardization in cellular manufacturing processes are still major challenges. In particular, when large numbers of cells (10^10^−10^12^) are required, conventional two-dimensional (2D) culture strategies, mainly based on manual, extremely space- and labour-intensive interventions, are practically and financially unsustainable [1–5].

In a scaling-up perspective and inspired by the manufacturing processes of therapeutics in biopharmaceutical industry [6,7], three-dimensional (3D) suspension culture has demonstrated to be an advantageous alternative to monolayer techniques for large-scale expansion of cells [4,5,8,9]. In detail, suspension methods have been widely adopted: (1) for scalable and controlled expansion of stem cells [10–15] and cancer cells [16–18]; (2) for guiding stem cell differentiation [13,19–22]; (3) for the production of cellular spheroids and tissue-like constructs [23–25]. The provision of a 3D suspension culture environment, mimicking the microenvironment of the cellular niche, has proven to be beneficial, promoting cell survival and retaining cell functional properties *in vitro* [9,26,27]. Moreover, when suspension is obtained by dynamic mixing of the culture medium, (1) the formation of gradients in, e.g., temperature, pH, dissolved oxygen, nutrients/metabolites is prevented, (2) the transport of oxygen and nutrients is increased, and (3) the sedimentation of cultured cells/constructs is avoided, thus going beyond the intrinsic limitations of static culture systems [4,7,9,28].

Nowadays, dynamic suspension culture for scalable production and differentiation of cells is mostly performed by stirred tank and rotating bioreactors [2,4]. Such devices are designed for providing a 3D homogenous culture environment and for enabling monitoring and control of culture parameters, leading to more reproducible, robust and cost-effective processes [5, 29,30,31]. However, most of these bioreactors still suffer from critical issues, limiting the upscaling and the standardization of the expansion bioprocesses. Concerning stirred tank bioreactors, their performance can be affected by (1) collisions of the cells with the impeller and (2) the onset of turbulent flow, that both can induce non-physiological mechanical and hydrodynamic-shear stresses on the cells and lead to cell damage. Moreover, these unfavourable conditions can affect cell growth rate and metabolism, interfere with stem cell pluripotency, and limit efficiency and reproducibility of the culture process [4,9,28,30,32,33]. Rotating bioreactors generate a low-shear stress culture environment, allowing to partially overcome the limitations of stirred tank devices. However, the complexity of the technological solutions adopted for rotation make these devices not easily scalable and unsuitable for continuous medium replacement and real-time monitoring [4].

We present here a versatile bioreactor suitable for tunable shear stress dynamic suspension cell culture. In detail, by adopting simple technological solutions and avoiding rotating components, the proposed bioreactor enables cell suspension by assuring a laminar mixing flow regime, thus guaranteeing oxygen and nutrient transport and ultimately homogeneous culture environment under a wide range of shear stress conditions.

In order to go beyond the experimental trial-and-error approach and to reach a deeper understanding of the fluid dynamics developing inside the culture environment [34,35], the design phase of the device has been supported by in silico multiphysics modelling, providing a comprehensive analysis of the operating principles of the bioreactor. Moreover, findings from the multiphysics simulations served as criteria to set the proper bioreactor operating conditions for preliminary *in vitro* tests. In particular, this first study was focused on assessing the suitability of the bioreactor as ultralow shear stress dynamic suspension device for cancer cell spheroid culture. To this purpose, the Calu-3 human lung carcinoma cell line was subjected to ultralow shear dynamic suspension provided by the device. The biological results indicate that this approach preserves cancer cell growth *in vitro*, including spheroid formation, and suggest the suitability of the proposed bioreactor for investigation on cell functional properties and for expansion of different cell types.

## Materials and Methods

### Dynamic suspension bioreactor

**Fig 1:**
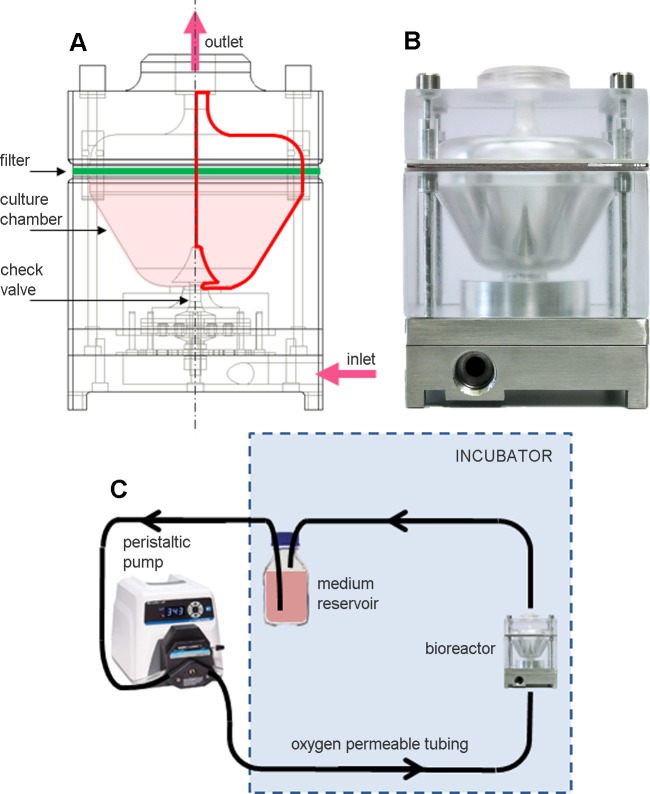
Dynamic suspension bioreactor. (A) Schematic draw of the bioreactor showing its internal components and its axial symmetry (red lines). (B) Picture of the bioreactor. (C) Schematic representation of the set-up of the bioreactor connected to the closed loop recirculation circuit and positioned within the incubator.

The design of the device (Fig 1A) was driven by two main requirements: (1) to provide dynamic suspension culture with proper mixing; (2) to guarantee a tunable ultralow-to-moderate shear stress culture environment, adjustable on the basis of culture requirements by simply modifying operating conditions. These objectives were achieved combining the peculiar geometric features of the bioreactor culture chamber with the continuous recirculation of the culture medium assured by a closed-loop recirculation circuit, avoiding the use of impellers and/or rotational components. This combination promotes the establishment of buoyant vortices within the culture chamber, that maintain cells/constructs in dynamic suspension, minimizing their sedimentation.

The bioreactor (Fig 1B, external dimensions = 95 mm x 70 mm x 70 mm) consists of: an AISI 316L stainless steel base; a polycarbonate culture chamber for housing the cells/constructs (chamber volume = 75 mL); a polycarbonate lid. The curvature and shape of the internal wall of the culture chamber were designed and optimized for the generation of buoyant vortices for specimen suspension (as detailed in the following). Suspended cells/constructs are confined inside the culture chamber by means of the presence of (1) an AISI 316L stainless steel unidirectional check valve (which prevents backflow and guarantees a symmetric flow inlet), and (2) a culture medium-permeable filter (Durapore^®^, MerckMillipore, Germany), which prevents accidental outputs of cells. The bioreactor is part of a closed loop circuit for the recirculation of oxygenated culture medium (Fig 1C). Such recirculation circuit is composed of a medium reservoir, oxygen-permeable peroxide-cured silicone tubing (Masterflex L/S^®^, Cole-Parmer, IL, USA) with quick-disconnect couplings, and a peristaltic pump (Masterflex L/S^®^, Cole-Parmer, IL, USA), for a total working volume of approximately 200 mL. To guarantee the adequate supply of oxygen within the culture chamber, the recirculation circuit was sized using an analytical oxygen mass balance model in accordance with Orr et al. [36].

The functioning principle of the bioreactor is based on the continuous recirculation of the culture medium inside the culture chamber under laminar flow regime, obtained through the modulation of the recirculation circuit flow rate, in order to produce from ultralow to moderate shear stress dynamic suspension conditions. In detail, the medium flows through the check valve, driven by the peristaltic pump against the static pressure gradient, and pervades the culture chamber. Successively, the medium passes through the filter and flows out from the lid, moving back to the reservoir in a continuous closed-loop process. The formation of buoyant vortices inside the culture chamber allows the dynamic suspension of the cultured cells/constructs (S1 Movie).

### Computational models

A computational multiphysics approach supported the design and the optimization phases of the device, allowing the identification of (1) the optimal geometry of the culture chamber, and (2) the operating conditions for dynamic suspension cell culture under defined shear stress values. A massive number of simulations was performed varying both cell/construct dimensions (in terms of their diameter) and highly-dilute cell inoculation densities, in order to study the sensitivity of the fluid flow to these culture parameters within the chamber volume.

Technically, taking advantage of the axial symmetry of the device (Fig 1A), a set of axisymmetric time-dependent numerical simulations was carried out using a customized finite volume technique-based commercial software (FLUENT, ANSYS Inc., PA, USA). The fluid domain was discretized using ICEM CFD software (ANSYS Inc., PA, USA). A mesh cardinality equal to 6.5x10^3^ quadrilateral cells was considered. As in previous studies [21,37], the concomitant presence of culture medium and cells was modelled using the Eulerian–Eulerian Multiphase Model, which allows mixtures of multiple separated yet interacting phases of a continuum to be described. For each phase the governing equations of motion, the Navier–Stokes equations, were solved by the numerical solver. The culture medium, considered as the primary phase, was assumed to be Newtonian with physical properties of culture media typically used in cell culture applications (dynamic viscosity = 1x10^-3^ Pa·s, density = 1000 kg/m^3^) [21]. Suspended cells, considered as the secondary immersed phase, were modelled as non-deformable spherical beads. In the explanatory example reported in this work, a density equal to 1070 kg/m^3^ [38] and an average diameter equal to 20 μm (i.e. the measured diameter of Calu-3 cancer cells) were considered. The presence of the filter was modeled as a porous medium characterized by a value of Darcy hydraulic resistance equal to 96x10^4^ m^-2^ for the culture medium and setting the maximum hydraulic resistance accepted by the solver (1x10^20^ m^-2^) for the cells, having the filter an average pore size of 5 μm, thus being impermeable to them. Cell inoculation was assumed to be uniform in the lower region of the culture chamber. This assumption was translated into the computational framework prescribing, as initial condition, a uniform volume fraction (VF) occupied by the cells (the secondary phase) in the lower vessel region (10 mL, in the explanatory example using Calu-3 cell line). Simulations were carried out considering always highly-dilute suspension cultures (Stokes numbers greatly lower than 1, VF value lower than 1%), for which variations in initial VF do not markedly affect the flow field of the primary phase. As an indicative limit value for sedimentation, a VF value higher than 20% was considered, corresponding to approximately one third of the maximum packing limit of 63%, i.e., the packing limit for non-deformable spherical beads regularly packed [21]. Simulations were extended over flow rate values in the range 5–120 mL/min, with a simulated culture time equal to 60 min, which was considered sufficient to fully describe the dynamics of the medium inside the culture chamber. The phase-coupled SIMPLE scheme was used for the pressure-velocity coupling. The Second Order Upwind and the QUICK formulation were used for the spatial discretization of the momentum and the secondary phase transport, respectively. Details related to model equations and boundary conditions are reported in S1 Text.

Moreover, in order to investigate the influence of the dynamic mixing establishing within the culture chamber on the evolution of physical and environmental quantities, the transport of a scalar quantity within the culture chamber was modelled. In detail, the transport of oxygen dissolved in the medium flowing inside the culture chamber was simulated by solving the advection/diffusion transport equation, coupled with Navier-Stokes equations. A fully anoxic culture medium inside the culture chamber was considered as initial condition (worst case). This computational model can be generalized to all the dissolved species characterized by similar Péclet numbers (i.e. the ratio between advective and diffusive transport rates). Details about model assumptions and equations are reported in S2 Text.

### *In vitro* cell culture

The performance of the bioreactor was explanatory tested in the ultralow shear stress dynamic culture frame (imposing a flow rate of 5 mL/min), as identified from the in silico analogue of the in vitro experiment (see Results). The Non Small Cell Lung Cancer (NSCLC) cell line Calu-3 (American Type Culture Collection, ATCC, VA, USA) was selected and the results of the dynamic culture were compared to a static suspension culture control. In detail, cells were grown in complete medium Dulbecco’s Modified Eagle Medium (DMEM, Sigma Aldrich, MO, USA) added with 10% Fetal Bovine Serum (FBS), 1% Penicillin/Streptomycin (P/S) and 1% Non-Essential Amino Acids (NEAA, Sigma Aldrich, MO, USA), and maintained under standard cell culture conditions at 37°C in a water-saturated atmosphere of 5% CO_2_ in air. Following expansion in cell culture flasks, 9x10^6^ Calu-3 cells (1.92x10^5^ cell/mL) were inoculated within the bioreactor culture chamber and cultured for 5 days in dynamic suspension with complete growth medium. The bioreactor was operated at a flow rate of 5 mL/min. In parallel, Calu-3 cells were seeded at the same density on low attachment culture flasks (Corning Inc., NY, USA) used as control, representing a model of static suspension culture. After 5 days, dynamic and static suspended cultured cells were rescued from the bioreactor and from the low attachment culture flask, respectively, and re-suspended in fresh growth medium for further analysis. Three independent static and dynamic suspension cultures were carried out.

### Assessments of *in vitro* cell culture

Calu-3 cells harvested from the bioreactor and from the low attachment culture flask and re-suspended in fresh growth medium were investigated by inverted microscope (Olympus CK40, Japan). Microphotographs were collected and analyzed by an image analysis software (Image Pro-plus 4.0, Media Cybernetics, USA) in order (1) to compute the number of single Calu-3 cells and the number of Calu-3 cells forming the spheroids, and (2) to determine the spheroid sizes.

Moreover, Calu-3 cells from dynamic and static suspended culture were processed for Transmission Electron Microscopy (TEM) analysis and for immunocytochemistry. For TEM analysis, Calu-3 cells were fixed in Karnovsky solution (4% formaldehyde, 5% glutaraldehyde). Samples were postfixed in 1% osmium tetroxide and dehydrated by increasing concentration of alcohol. Then, samples were washed with propylene oxide and embedded in epoxy resin. Sections of 0.5 μm thickness were stained with methylene blue and safranin to morphologically select the field of interest. Subsequently, ultrathin sections were collected on a 300-mesh copper grid and, after staining with uranyl acetate and lead citrate, were qualitatively examined under TEM (Philips EM 208S, The Netherlands). To evaluate the fraction of cells in active cell cycle, the presence of reversible DNA double strand breaks, and the apoptotic cell death, cells were fixed with 4% paraformaldehyde and cytocentrifuged on a glass slide to obtain a density of 10^5^ cells per spot. Cell spots were stained by anti-Ki67 (Ki67, mouse monoclonal, DAKO, Italy) and anti-gamma histone H2AX (γH2AX, rabbit polyclonal, Bethyl Laboratories, TX, USA) antibodies and revealed by DAB (3,3’-diaminobenzidine) Peroxidase (HRP) Substrate Kit reaction (DAKO, Italy). The quantitative assessment of the fraction of Ki67 and γH2AX positive cells was carried out by computing the number of positive nuclei over a total of 900–2000 nuclei counted on each analyzed sample. The apoptotic cell death at the single-cell level was investigated by using the In Situ Cell Death Detection Kit, Fluorescein assay (Enzyme Solution TdT, Label Solution fluorescein-dUTP, Roche). Green nuclear fluorescence positivity was measured using Olympus microscope BX60. Nuclei were recognized by the blue fluorescence of 4’,6-diamidine-2-phenyndole (DAPI, Sigma, Italy). The fraction of dead cells was assessed by counting the number of apoptotic nuclei over a total of nearly 1000 cells. Data were analyzed using the one-way ANOVA test. Results were considered statistically significant when p<0.05. In order to investigate the possible accidental adhesion of cells/spheroids on the filter after 5 days of dynamic culture within the bioreactor, the filter was fixed with 4% paraformaldehyde and incubated with DAPI for 15 minutes at room temperature, and successively investigated by fluorescence microscope to assess the presence of nuclei on its surface. Finally, in order to assess the presence of adhered Calu-3 cells and sedimentation in the lower region of the culture chamber, after cell rescue the internal wall of the culture chamber was rasped by a cell scraper and washed with Phosphate Buffered Saline (PBS). The PBS was therefore collected within a Petri dish and observed by inverted microscope to detect the presence of Calu-3 cells.

## Results

### Flow dynamics within the bioreactor culture chamber

**Fig 2:**
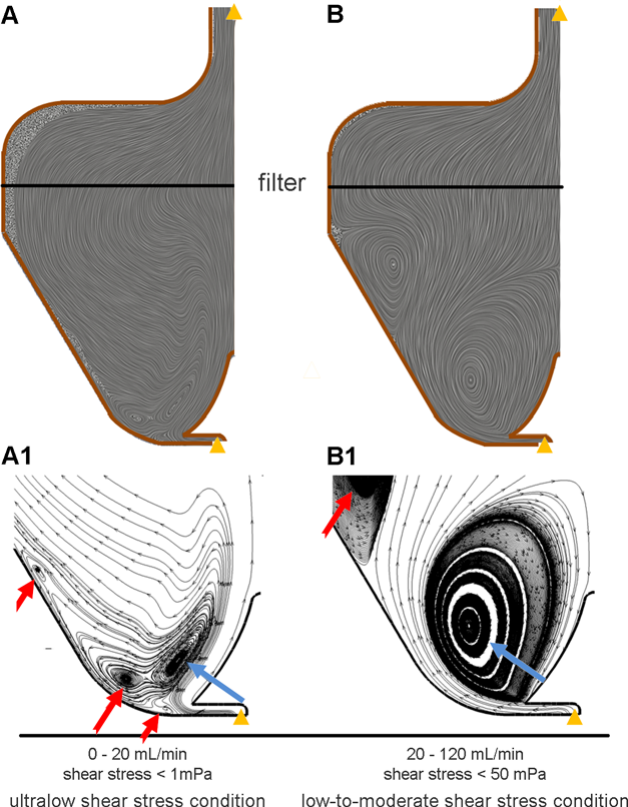
Flow field within the bioreactor. Flow field visualization of the mutual interaction between the medium (primary phase) and the cells/constructs (dispersed phase) within the culture chamber for ultralow (A and A1) and low-to-moderate (B and B1) shear stress conditions. Flow field is depicted using both linear integral convolution lines (A and B), and a classical streamline representation (A1 and B1). Yellow arrows indicate the flow inlet and outlet. Blue arrows indicate the primary buoyant vortices. Red arrows indicate the secondary vortices.

**Fig 3:**
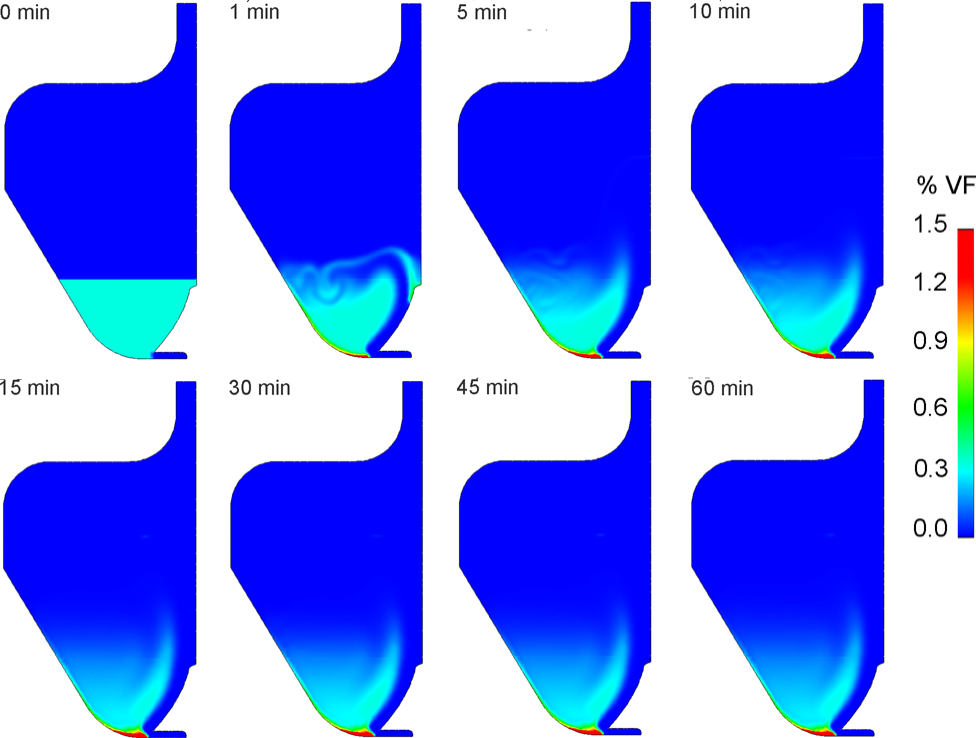
Temporal evolution of the volume fraction occupied by suspended cells inside the bioreactor culture chamber. Contour plots of the temporal evolution of the VF occupied by the suspended cells inside the bioreactor culture chamber from 0 to 60 min of simulated time, with an imposed flow rate of 5 mL/min (ultralow shear stress condition, similarly to the experimental in vitro test) and 9x10^6^ inoculated cells (initial VF = 0.48%). After a transient of about 5 min, the 95.3% of the inoculated cells are suspended at an average VF value of approximately 0.33%, very close to the initial VF value. At the bottom of the culture chamber, a small volume of about 194 μL is characterized by a VF value around 6%, more than three times lower than the set threshold value of sedimentation (20%), which dynamically involves only the 2% of the inoculated cells.

**Fig 4:**
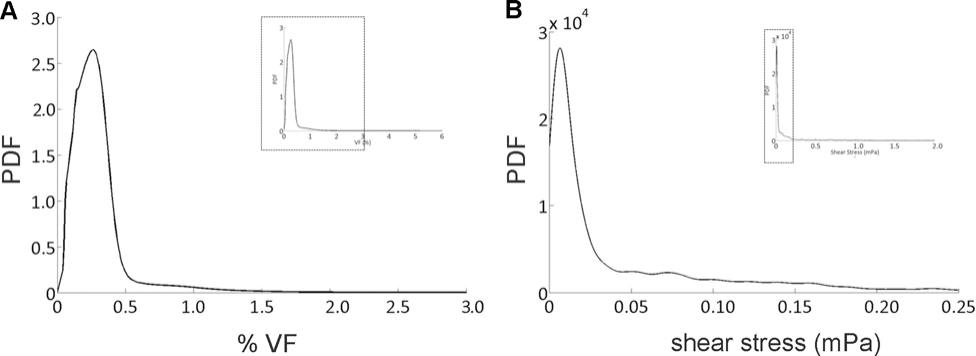
Probability density functions of volume fraction and shear stresses. Probability density functions (PDF) of cell VF (A) and shear stresses (B) values experienced by the cellular phase within the bioreactor culture chamber after 60 min, with an imposed flow rate of 5 mL/min and 9x10^6^ inoculated cells.

Multiphysics numerical simulations allowed to characterize the flow field inside the bioreactor culture chamber. Fig 2 depicts diagrammatic representations of the typical medium flow structures establishing inside the culture chamber, resulting from the mutual interaction between the medium (primary phase) and the cells/constructs (dispersed phase), depending on the imposed flow rate. In detail, in case of flow rate values lower than 20 mL/min (Fig 2A and 2A1), the medium streaming into the culture chamber through the valve has not sufficient energy to markedly interact with the side wall of the culture chamber. The balance between hydrodynamic and gravitational forces leads to the formation of a dynamic big buoyant vortex located far from the wall of the chamber (Fig 2A1). This buoyant vortex is surrounded by smaller vortical structures located closer to the wall, which assure the suspension of the cultured cells and increase mixing and transport (Fig 2A1). As an example, Fig 3 shows the time evolution of the VF occupied by suspended cells inside the culture chamber, obtained simulating the presence of 9x10^6^ inoculated cells (initial VF = 0.48%) and imposing a flow rate value of 5 mL/min (ultralow shear stress condition, similarly to the experimental in vitro test). It can be observed that cultured cells are maintained mostly uniformly distributed in the lower region of the culture chamber. In detail, after a transient of about 5 min, the 95.3% of the inoculated cells are suspended at an average VF value of approximately 0.33%, which is close to the initial VF value (0.48%), with the peak of probability density function (PDF) value equal to 2.5, corresponding to VF values between 0 and 0.5% (Fig 4A). At the bottom of the culture chamber, a small volume of about 194 μL is characterized by a VF value around 6%, which dynamically involves only the 2% of the inoculated cells (Fig 3). This packing value is more than three times lower than the threshold value of sedimentation that was set (20%) and about ten times lower than the maximum packing limit of 63%. Notably, when a flow rate lower than 20 mL/min is adopted, the distribution of shear stress values experienced by the cells within the culture chamber reveals that the highest shear stress levels are lower than 1 mPa (Fig 4B), with mean and median values close to 1x10^-2^ mPa (the so called ultralow shear stress condition).

Increasing the flow rate beyond 20 mL/min promotes the occurrence of Coanda effect [39] within the bioreactor culture chamber: the jet entering the culture chamber is attracted to the nearby wall and, due to the peculiar wall curvature, a separation region occurs far from the bottom wall of the chamber (Fig 2B and 2B1). As a result, a large clockwise buoyant vortex, which counterbalances the gravitational force and thus maintains cells/constructs in suspension, is generated (Fig 2B1). Near the outer wall, a further smaller vortex develops, which can play the beneficial role of enhancing the mixing and the suspension of floating constructs (Fig 2B1). Adopting such a flow rate range (30–120 mL/min), skewed right shear stress distributions were obtained, with mean values ranging from 2 to around 7 mPa, with peak shear stress values within the culture chamber lower than 50 mPa (low-to-moderate shear stress condition, see details in S3 Text).

Concerning the transport of dissolved oxygen within the culture chamber imposing a fully anoxic initial condition for the medium, the numerical simulation clearly shows that within 840 s (14 minutes) the partial pressure of the dissolved oxygen is replenished in more than the 90% of the culture chamber volume (S2 Movie, S2 Text). The obtained results can be generalized to the transport of other species dissolved in the culture medium, because their transport is characterized by Péclet numbers two-three orders of magnitude higher than unity, confirming that fluid structures establishing inside the chamber promote transport of dissolved oxygen and nutrients through mixing, thus homogenizing their concentration.

### *In vitro* culture outcome

**Fig 5:**
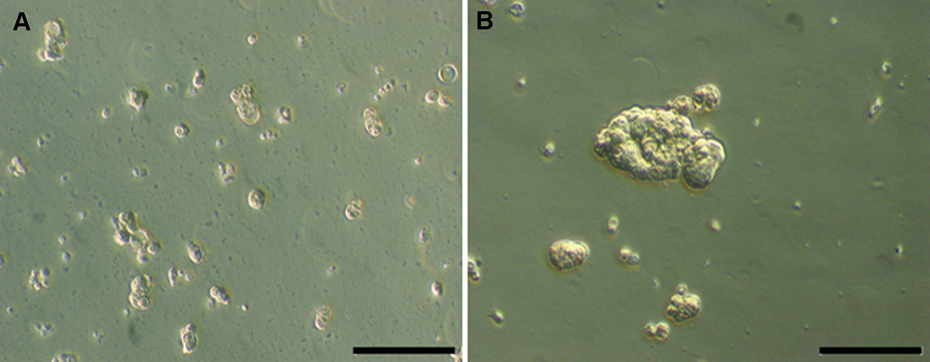
Morphological comparison by phase contrast microscopy. After 5 days of suspension culture, (A) Calu-3 cells cultured in static suspension show individual cells or very small clusters, (B) Calu-3 cells cultured under dynamic suspension show the formation of spheroids. Scale bars 200 μm.

After 5 days of suspension culture, cells were rescued from the low attachment culture flask (static suspension) and from the bioreactor culture chamber (dynamic suspension). Firstly, they were morphologically analyzed: observed by phase contrast microscopy, Calu-3 cultured under static suspension show individual cells or very small clusters (Fig 5A), while cells cultured within the bioreactor under dynamic suspension clearly show the formation of spheroids (Fig 5B). In particular, the ratio between Calu-3 cells forming the spheroids and single Calu-3 cells is 59.7 for Calu-3 cells harvested from the low attachment culture flask and 76.0 for Calu-3 cells cultured within the bioreactor.

**Fig 6:**
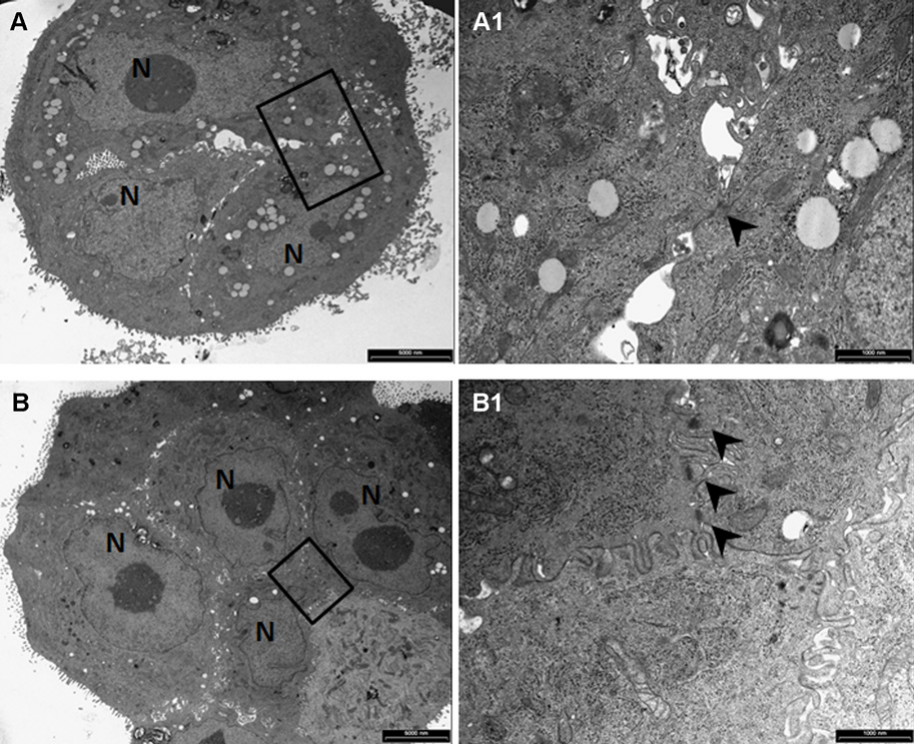
Ultrastructural comparison by TEM. The TEM images show (A) a small cluster (3 cells) of Calu-3 cells grown in static suspension, and (B) a larger spheroid (9 cells) of Calu-3 cells cultured within the bioreactor, harvested both after 5 days of suspension culture. Prominent nucleoli (N: nuclei), cytoplasmic structures and longitudinally and transversally oriented microvilli are characteristic features of NSCLC cell line Calu-3. High magnification views of areas included in black rectangles in panels A and B shown, respectively, (A1) a single tiny adherence junction (arrowhead) among cells cultured under static suspension, and (B1) several well-developed adherence junctions (arrowheads) developed by Calu-3 cultured within the bioreactor. Scale bars: A and B = 5 μm; A1 and B1 = 1 μm.

Moreover, ultrastructural analysis by TEM allows to observe that Calu-3 from static suspension are partially connected by weak and tiny adherence junctions (Fig 6A and 6A1), with morphological alterations (S1 Fig). Conversely, the spheroids harvested from the bioreactor culture chamber are 2.4-fold (p<0.001) larger (average area = 23699±5645 μm^2^) than clusters rescued from the low attachment culture flask (average area = 9787±2202 μm^2^), and are composed of cells characterized by the typical morphological features of Calu-3, such as prominent nucleoli and membranes microvilli (Fig 6B), with well-developed adherence junctions (Fig 6B1).

**Fig 7:**
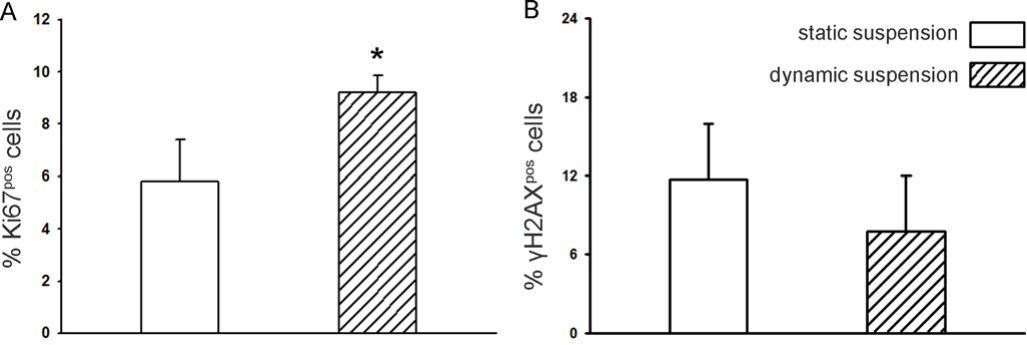
Quantitative comparison of cycling cells and double DNA strand breaks. (A) Bar graph of the measurement of Ki67 positive cells, showing the fraction of cycling Calu-3 cells after static and dynamic suspension culture (*: p<0.05 vs static suspension). (B) Bar graph of the measurement of γH2AX positive cells, quantifying the double DNA strand breaks in Calu-3 cells harvested from static and dynamic suspension culture.

These observations are supported by the assessment of Ki67 immunostaining, which indicates that the fraction of cycling Calu-3 cells is significantly higher (1.58-fold increase) when cultured in dynamic rather than in static suspension conditions (Fig 7A). Furthermore, from the quantification of the DNA double strand breaks, it is possible to note a downward trend (1.5-fold reduction, even if not statistically significant) in the fraction of γH2AX^pos^ Calu-3 cells cultured within the bioreactor compared to the cells cultured under static suspension (Fig 7B). This is confirmed by the apoptotic cell death assay, indeed the fraction of apoptotic Calu-3 cells harvested from the static suspension control (46.5 ± 4.5%) was 16.9-fold higher than cells harvested from the bioreactor (2.7 ± 0.2%), (p<0.05).

As regards (1) the possible accidental adhesion of cells/spheroids on the filter, and (2) the cell sedimentation in the lower region of the culture chamber after 5 days of dynamic culture within the bioreactor, fluorescence analysis showed that no cells adhered to the filter, and no cells were detected within the PBS collected from the scraped lower region of the chamber, confirming that no sedimentation occurred.

## Discussion

In this study, a versatile bioreactor for culturing cells in dynamic suspension is presented. Due to the combination of the peculiar shape of the culture chamber with the continuous recirculation of the culture medium within a closed-loop circuit, this bioreactor enables laminar dynamic suspension culture at tunable ultralow-to-moderate shear stress values. Avoiding the use of impellers and/or rotational components, the presented device overcomes some major limitations of the current dynamic suspension methods. In fact, it is well established that within the stirred systems (e.g., spinner flasks, stirred tank bioreactors) (1) the interaction of cells with the moving components, and (2) the complex fluid dynamics, characterized by turbulence and/or detrimental shear stresses, could lead to cell damage and consequent low expansion efficiency and limited bioprocess reproducibility [4,9,28,30,32,33]. Differently, rotating bioreactors provide laminar, low-shear stress culture environments, but the complex technological solutions needed to impart rotation make them not easily scalable and unsuitable for continuous medium replacement and real-time monitoring [4]. In this context, an impeller-free dynamic suspension bioreactor, characterized by laminar, ultralow-to-moderate shear flow within the culture chamber and based on simple technological solutions, has been (1) designed, (2) characterized and optimized by means of computational multiphysics, (3) prototyped, and (4) experimentally tested for dynamic suspension cell culture.

More in detail, multiphysics modelling allowed to optimize the design of the device in terms of its performance in establishing dynamic suspension of biological specimens at low shear stress levels. By selecting the operating flow rate and exploiting the geometric features of the culture chamber, the device enables to provide dynamic cell suspension conditions at different shear stress levels, from ultralow (less than 1 mPa) to moderate (less than 50 mPa) values. The computational modelling allowed to define two main flow conditions for culturing cells: (1) the ultralow shear stress condition, obtained working with flow rates in a range up to 20 mL/min (Fig 2A and 2A1); and (2) the low-to-moderate shear stress condition, which can be established working with flow rates in the range 20–120 mL/min, characterized by the formation of larger suspension buoyant vortices (Fig 2B and 2B1). Adopting flow rates under 20 mL/min, shear stress values lower than 1 mPa develop within the culture chamber (ultralow shear stress condition, Fig 4B), while increasing the flow rates up to 120 mL/min, skewed right shear stress distributions are obtained, with mean values ranging from 2 to around 7 mPa (low-to-moderate shear stress condition, details in S3 Text). The (tunable) shear stress values produced by this dynamic suspension bioreactor are (1) one order of magnitude lower than the shear stress values normally developing within a commercial spinner flask where, imposing agitation rates ranging from 15 to 50 rpm, mean shear stress values ranging from 20 to around 120 mPa are reached (with peak values of 200 mPa) [40], and (2) some orders of magnitude lower than the reference shear stress value considered critical (250 mPa) for sensitive cells like human embryonic stem cells or neonatal rat cardiomyocytes [33]. Furthermore, the simulated transport of oxygen dissolved in the medium confirms that the presence of laminar, dynamic vortex structures within the culture chamber promotes nutrients and gases mixing and transport, as well as cell transport during dynamic suspension, guaranteeing their homogeneous distribution.

A lung tumour-derived epithelial cell line (Calu-3) was selected for the preliminary tests under ultralow shear stress conditions because of the property to form multicellular spheroids, typically used for investigation of lung cancer biology and ontogeny of epithelial tissues in vivo [41]. The biological findings coming from the culture of Calu-3 cells in ultralow shear stress dynamic suspension confirm that with the use of the presented device (1) suspension is ensured (no sedimentation was observed), (2) the formation of functional 3D cell spheroids with active intercellular connection is promoted (Figs 5 and 6), and (3) a culture environment is established that, in comparison to the static suspension control, increases spheroid size and cycling cell number, and reduces the double strand DNA damage (Fig 7).

Some limitations could weaken the potential of the presented bioreactor in culturing cells in dynamic suspension. As the bioreactor is at a prototypal stage, the operating flow rates are currently manually set through the peristaltic pump of the recirculation circuit. However, a control system for process automation can be easily integrated in the loop. Moreover, direct sampling and/or monitoring are currently not feasible during bioreactor functioning. In the future, the recirculation circuit will be equipped with specific sensors, upstream and downstream the bioreactor chamber, in order to provide real-time information about the metabolic behaviour of cultured cells. Concerning the computational multiphysics approach, a main limitation is that the aggregation and disaggregation of the cultured cells/constructs are not considered in the model, since the biological sample size was assumed to be always equal to the initial cell dimension (20 μm). This choice was dictated by a primary interest in assessing the fluid dynamics inside the culture chamber at the very early stage of the culture process, when it is fundamental to ascertain the suspension/sedimentation of the cells, and giving indications on the initialization of the experimental procedure. Therefore, since aggregation and disaggregation phenomena have typical characteristic time of days, they were neglected in the simulation provided in this paper. Moreover, cell growth has not been included in the numerical model since it was assumed that for the time interval considered for the simulation, it could be neglected.

Although these limitations could weaken the findings of this study, the herein presented combination of outputs of the in vitro experiment and the corresponding in silico simulation has demonstrated the potential of the device in culturing cells in 3D dynamic suspension at low shear stresses. In particular, the a priori knowledge (from simulations) on the flow environment inside the bioreactor culture chamber employed for culturing Calu-3 cells in dynamic suspension allowed to obtain a more favourable condition to cancer cells aggregation than the static suspension control.

In conclusion, here we propose a suspension bioreactor design, conceived to create a unique fluid dynamic environment inside the culture chamber avoiding any moving component. By adopting simple technological solutions, the presented versatile bioreactor allows to culture specimens of different dimensions in laminar, dynamic suspension over a range of shear stress conditions, finally allowing to overcome major limitations of the current dynamic suspension devices [4,9,28,30,32,33].

In the future, such a device could be considered to be used: (1) as model system, for investigating the influence of dynamic suspension conditions on functional properties of different types of cells/constructs; (2) as aggregation system, for culturing and investigating cell spheroids; (3) as expansion and differentiation system, e.g., for expansion and differentiation of stem cells, for which non-physiological shear stress values can affect maintenance of pluripotency and interfere with lineage-specific differentiation, thus providing a low-shear culture condition that could significantly increase the bioprocess efficiency and reproducibility.

## Supporting Information

S1 Fig Morphological alterations of Calu-3 cells cultured under static suspension. The TEM images of Calu-3 cells cultured under static suspension conditions show (A) the presence of both several autophagosomes (white arrowheads) in a cell with preserved ultrastructure and severe depletion of cytoplasmic and nuclear (N) structures in a nearby cell; (B) the partial loss of cytoplasmic organelles (*) together with the formation of large vacuoles (#). Scale bars 5 μm.
(TIF)

S1 Movie Dynamic suspension within the bioreactor. The movie shows the formation of the buoyant vortices within the bioreactor culture chamber that maintain in dynamic suspension the cultured cells/constructs when the bioreactor is connected to the closed loop recirculation circuit.
(MP4)

S2 Movie Evolution in time of the dissolved oxygen within the culture chamber. The movie shows the evolution in time of the dissolved oxygen partial pressure (mmHg) inside the culture chamber, imposing a fully anoxic initial condition. It is possible to note that after 840 s (14 minutes) the 90% of the culture chamber is completely saturated of oxygen.
(MP4)

S1 Text Computational model equations and boundary conditions. (DOCX)

S2 Text Evaluation of dynamic mixing: Dissolved oxygen mass transport model. (DOCX)

S3 Text Shear stress distributions imposing 30–120 mL/min flow rates. (DOCX)
