# Correction: A Versatile Bioreactor for Dynamic Suspension Cell Culture. Application to the Culture of Cancer Cell Spheroids

**DOI:** 10.1371/journal.pone.0157289

**Published:** 2016-06-03

**Authors:** The PLOS ONE Staff

There are errors in the Author Contributions. The correct contributions are: Conceived and designed the device: DMassai GC GI DG UM. Conceived and performed the computational multiphysics modeling: GI DMassai UM DG. Conceived and performed the biological tests: DMadeddu AF CF FQ DMassai GC. Analyzed the computational multiphysics modeling data: GI DMassai UM DG MAD. Analyzed the biological data: DMadeddu CF AF FQ. Contributed reagents/materials/analysis tools: FQ AA GFDL UM. Conceived and wrote the manuscript: DMassai GI UM DMadeddu FQ. The publisher apologizes for the errors.
