# Supplementary figures and images for: A Versatile Bioreactor for Dynamic Suspension Cell Culture. Application to the Culture of Cancer Cell Spheroids
Source: PLoS One. 2016 May 4;11(5):e0154610. doi: 10.1371/journal.pone.0154610 (PMC4856383; doi:10.1371/journal.pone.0154610)

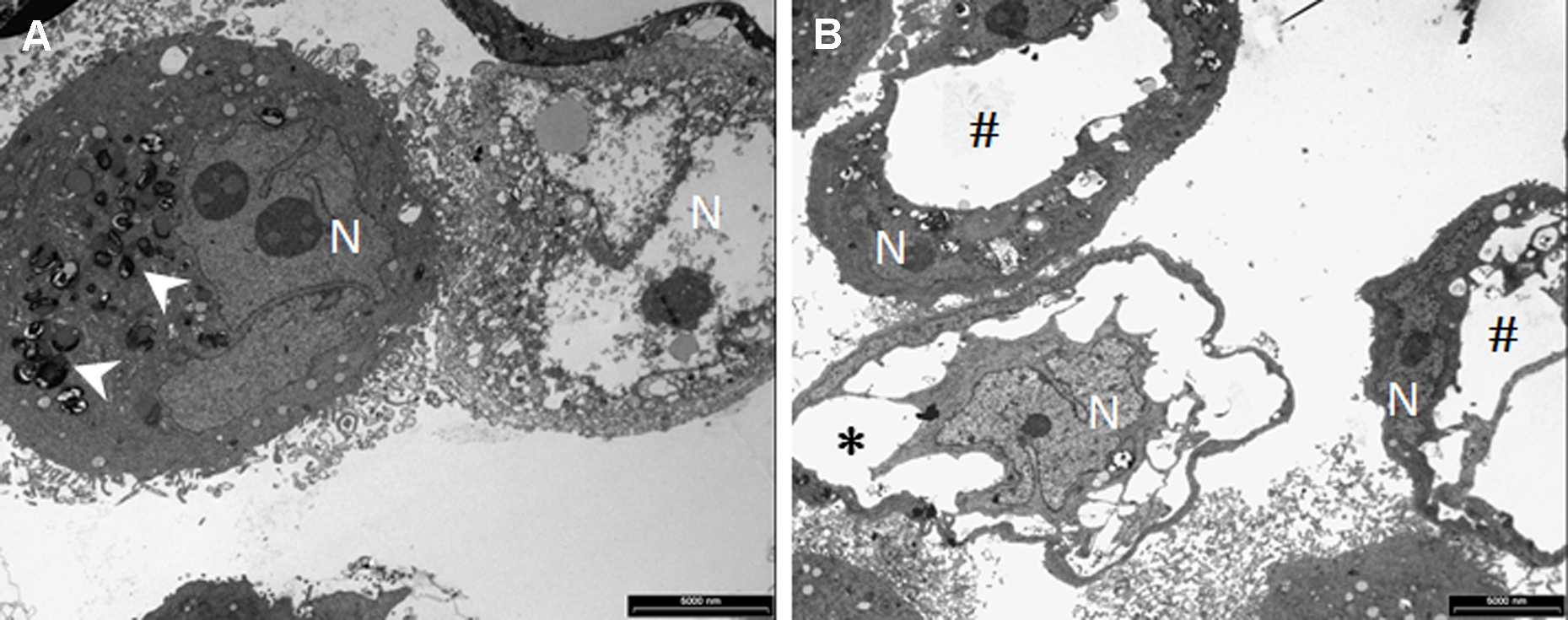

Supplement: S1 Fig — The TEM images of Calu-3 cells cultured under static suspension conditions show (A) the presence of both several autophagosomes (white arrowheads) in a cell with preserved ultrastructure and severe depletion of cytoplasmic and nuclear (N) structures in a nearby cell; (B) the partial loss of cytoplasmic organelles (*) together with the formation of large vacuoles (#). Scale bars 5 μm. (TIF) [file pone.0154610.s001.tif]
