# Supplementary material for: A Versatile Bioreactor for Dynamic Suspension Cell Culture. Application to the Culture of Cancer Cell Spheroids
Source: PLoS One. 2016 May 4;11(5):e0154610. doi: 10.1371/journal.pone.0154610 (PMC4856383; doi:10.1371/journal.pone.0154610)
Supplement: S1 Text — (DOCX) [file pone.0154610.s004.docx]

**S1 Text. Computational model equations and boundary conditions.**

The concomitant presence of culture medium and suspended cells was modelled by means of an Eulerian-Eulerian multiphase model. In our model both phases are modelled as interacting phases of a continuum. For both phases Navier-Stokes equations are solved by the numerical solver. Subscripts p and s refer to primary (i.e. culture medium) and secondary (i.e. suspended cells) phases, respectively. By neglecting the mass transfer between the two phases, Navier-Stokes equations can be written in the form:

*Continuity equation*

$\frac{\partial}{\partial t}(\rho_{p}VF_{p})+\nabla\cdot(\rho_{p}VF_{p}\overrightarrow{v_{p}})=S_{p}$ Eq.1

*Conservation of momentum*

$\frac{\partial}{\partial t}(\rho_{p}VF_{p}\overrightarrow{v_{p}})+\nabla\cdot(\rho_{p}VF_{p}\overrightarrow{v_{p}}\overrightarrow{v_{p}})=-VF_{p}\nabla P+\nabla\cdot\overline{\tau}_{p}+\rho_{p}VF_{p}\overrightarrow{g}+\sum_{P=1}^{n} (\overrightarrow{R}_{sp}+\overrightarrow{W}_{p}+\overrightarrow{W}_{vm,p})$ Eq.2

where *VF_p_* is the volume fraction, *ρ_p_* the density and $\overrightarrow{v_{p}}$is the velocity of the *p-th* phase; *S_p_* is the mass-source term, is the stress-strain tensor of the primary phase p; $\overrightarrow{W}_{p}$ is the external body force, $\overrightarrow{W}_{vm,p}$ is the virtual mass force (which were neglected in our model); and $\overrightarrow{R}_{sp}$ is the interaction force between phase *p* and secondary phase *s* such as drag force which primary phase exerts on granular immersed secondary phase (according to [1]). P is the pressure shared by the two phases. The same equation can be written identical for secondary phase *s*, as:

*Continuity equation*

$\frac{\partial}{\partial t}(\rho_{s}VF_{s})+\nabla\cdot(\rho_{s}VF_{s}\overrightarrow{v_{s}})=S_{s}$ Eq.3

*Conservation of momentum*

$\frac{\partial}{\partial t}(\rho_{s}VF_{s}\overrightarrow{v_{s}})+\nabla\cdot(\rho_{s}VF_{s}\overrightarrow{v_{s}}\overrightarrow{v_{s}})=-VF_{s}\nabla P+\nabla\cdot\overline{\tau}_{s}+\rho_{s}VF_{s}\overrightarrow{g}+\sum_{P=1}^{n} (\overrightarrow{R}_{ps}+\overrightarrow{W}_{s}+\overrightarrow{W}_{vm,p})$ Eq.4

To solve Equations 1-4, the phase-coupled SIMPLE scheme was applied for the pressure-velocity coupling. The Second order upwind and the QUICK formulation were applied for the spatial discretization of the momentum and the secondary phase transport, respectively. A time step equal to 0.002 s was set in order to reach the numerical stability. Absolute convergence criterion was applied and convergence was considered achieved for relative residuals below 10^-6^ for Navier-Stokes equations and 10^-7^ for volume fraction residual. Flow rate value was imposed at the inflow section of the chamber in terms of flat velocity profile. At the chamber outlet a null pressure gauge condition was prescribed. No-slip condition was applied at the walls.

References

1. Gidaspow D, Bezburuah R and Ding J. Hydrodynamics of circulating fluidized beds, kinetic theory approach. In Fluidization VII, Proceedings of the 7th Engineering Foundation Conference on Fluidization. 1992;75-82 .
