# Supplementary material for: A Versatile Bioreactor for Dynamic Suspension Cell Culture. Application to the Culture of Cancer Cell Spheroids
Source: PLoS One. 2016 May 4;11(5):e0154610. doi: 10.1371/journal.pone.0154610 (PMC4856383; doi:10.1371/journal.pone.0154610)
Supplement: S2 Text — (DOCX) [file pone.0154610.s005.docx]

**S2 Text. Evaluation of dynamic mixing: Dissolved oxygen mass transport model.**

In order to investigate the influence of the dynamic mixing establishing within the bioreactor culture chamber on the evolution of physical and environmental quantities (e.g., nutrients and dissolved gases), the transport of a scalar quantity within the culture chamber was modelled. In detail, the advection/diffusion transport equation was solved to simulate the transport of oxygen dissolved in the medium flowing inside the culture chamber:

$\frac{\partial}{\partial t}(\rho Y_{O_{2}})+\nabla\cdot(\rho\vec{v}Y_{O_{2}})=\rho D_{O_{2}}\nabla^{2}Y_{O_{2}}$ Eq. 1

where *Y_O2_* is the dissolved oxygen mass fraction (i.e. the mass of the species per unit of mass of the culture medium in which oxygen is dissolved), and *D_O2_* is the oxygen diffusivity within the culture medium (2.55 x10^-9^ m^2^/s). The advection/diffusion equation for dissolved oxygen was coupled, by means of the advective velocity field of the culture medium $\vec{v}$, with the Navier-Stokes equations, considering the culture medium as a continuum:

$\nabla\cdot\overrightarrow{v}=0$ Eq. 2

$\frac{\partial}{\partial t}(\rho\overrightarrow{v})+\nabla\cdot(\rho\overrightarrow{v}\overrightarrow{v})=-\nabla P+\nabla\cdot\overline{\tau}+\rho\overrightarrow{g}$ Eq. 3

where *ρ* is the culture medium density, $\overrightarrow{v}$is the culture medium velocity, and is the stress tensor.

A constant inlet flow rate value (40 mL/min, intermediate value in the functioning range of the bioreactor) of oxygen-saturated culture medium (partial pressure of dissolved oxygen = pO_2_ = 159 mmHg) was imposed at the inlet port. Fully anoxic medium in the culture chamber was considered as initial condition (pO_2_ = 0 mmHg, worst case). This setting allowed to calculate the so called ‘blend time’, i.e., the time required to achieve a predefined level of homogeneity of a tracer in a mixing vessel.

Simulations were performed using a finite-volume-based commercial code (Fluent, ANSYS Inc., PA, USA). The Second Order Upwind formulation was used to solve the momentum and oxygen transport equation.

The simulated transport of dissolved oxygen over time through the culture chamber is presented in S2 Movie in terms of dissolved oxygen partial pressure (pO_2_). It can be noticed that, imposing an initial fully anoxic condition within the culture chamber (worst case), the pO_2_ is replenished in more than the 90% of the culture chamber volume in 840 s (14 minutes). Spatial gradients still persist in small regions of the culture chamber where cells/constructs are not present, but in longer times oxygen will be transported also in those regions by diffusion. The obtained results confirm that the hydrodynamic structures establishing within the culture chamber promote transport of dissolved oxygen mainly through advective mixing, thus homogenizing its concentration and minimizing spatial gradients.
