# Supplementary material for: A Versatile Bioreactor for Dynamic Suspension Cell Culture. Application to the Culture of Cancer Cell Spheroids
Source: PLoS One. 2016 May 4;11(5):e0154610. doi: 10.1371/journal.pone.0154610 (PMC4856383; doi:10.1371/journal.pone.0154610)
Supplement: S3 Text — (DOCX) [file pone.0154610.s006.docx]

**S3 Text. Shear stress distributions imposing 30-120 mL/min flow rates.**

In this section the distribution, in terms of probability density function (PDF), of the shear stress values developing within the bioreactor culture chamber when working flow rates higher than 20 mL/min are adopted is quantitatively described. The shear stress values were calculated by means of the multiphysics numerical model defined in the Materials and Methods Section. In Figure S3 Text, the low-to-moderate shear stress distributions related to four different flow rates ( i.e., 30, 50, 70 and 120 mL/min) are shown. It can be observed that the distributions of shear stress values are markedly skewed right and characterized by mean and mode values consistently below the reference shear stress limit of 250 mPa [32]. Indeed, mean shear stress value inside the culture chamber range from approximately 2 to 7 mPa (for 30 and 120 mL/min, respectively), while mode values are lower than 1 mPa for each reported flow rate.





**Figure S3 Text. PDF of shear stress values within the bioreactor culture chamber at four different flow rates (30, 50, 70, and 120 mL/min).**
